# Membrane-Free Stem Cell Components Inhibit Interleukin-1α-Stimulated Inflammation and Cartilage Degradation In Vitro and In Vivo: A Rat Model of Osteoarthritis

**DOI:** 10.3390/ijms20194869

**Published:** 2019-09-30

**Authors:** Ho Jeong Lee, Seon Min Lee, Yeon Gyu Moon, Yeon Seop Jung, Ju Hong Lee, Venu Venkatarame Gowda Saralamma, Young Sil Kim, Jung Eun Pak, Hye Jin Lee, Gon Sup Kim, Jeong Doo Heo

**Affiliations:** 1 Biological Resources Research Group, Gyeongnam Department of Environment Toxicology and Chemistry, Korea Institute of Toxicology (KIT), 17 Jeigok-gil, Jinju 52834, Koreasmlee84@kitox.re.kr (S.M.L.); ygmoon@kitox.re.kr (Y.G.M.); yeonseop.jung@kitox.re.kr (Y.S.J.); juhonglee@kitox.re.kr (J.H.L.); 2 Research Institute of Life science and College of Veterinary Medicine, Gyeongsang National University, 501 Jinju-daero, Jinju 52828, Korea; gowdavenu27@gmail.com; 3 T-Stem Co., Ltd. Changwon, Gyengsangnam-do 51573, Korea; tstem@t-stem.com (Y.S.K.); rari0713@t-stem.com (J.E.P.); hyejin_1216@t-stem.com (H.J.L.)

**Keywords:** NF-kB/MAPKs pathway, stem cells, anti-inflammatory, anti-osteoarthritis, rat chondrocytes

## Abstract

Membrane-free stem cell components (MFSCC) from basal adipose tissue-derived stem cells (ADSCs) are unknown for the treatment strategies in osteoarthritis (OA). OA has been considered to be associated with inflammatory damage and cartilage degradation. In this study, we intended to investigate the molecular mechanism of the anti-inflammation and cartilage protection effect of MFSCC in vitro (rat primary chondrocytes) and in vivo (rat OA model). The MFSCC treatment significantly inhibited interleukin-1α (IL-1α) stimulated inflammation and cartilage degradation. The MFSCC considerably reduced the levels of inflammatory factors such as iNOS, COX-2, NO, and PGE_2_ and was suppressed NF-κB and MAPKs signaling pathways in IL-1α-stimulated rat chondrocytes. Additionally, biomarkers of OA such as MMP-9, COMP, and CTX-II decreased in the monosodium iodoacetate (MIA)-induced rat OA model by MFSCC treatment. In conclusion, the MFSCC was established to suppress IL-1α induced inflammation and cartilage degradation in vitro and in vivo. These findings provide new insight for understanding OA therapy using membrane-free stem cell approaches.

## 1. Introduction

Osteoarthritis (OA), an immensely prevalent cartilage degenerative disorder causing pain and functional impairment, represents an enormous societal and economic burden in the world [1]. OA is now a well-established disease that affects all joint tissues, illustrated by the gradual degeneration of the articular cartilage, subchondral bone remodeling, vascular invasion of the articular surface, and synovial inflammation (synovitis) [2]. OA, being an age-related disease, affects 240 million people globally, including about 18% of women and 10% of men in the aged population (60 years old), and OA is expected to be the single greatest cause of disability in the common population by 2030 [3]. However, due to the lack of effective treatment for OA, a large population of patients certainly need joint replacement, causing substantial economic and social concerns. The imbalance between catabolic and anabolic factors in joints and inflammation causes pervasive cartilage damage, which is crucial to the progression of OA. Increasing evidence suggests that synovial inflammation is correlated with the pathogenesis and progression of OA, and local inflammatory responses plays a decisive role in the OA development [4].

Elevated levels of pro-inflammatory cytokines interleukin-1β (IL-1β), interleukin-6 (IL-6) and tumor necrosis factor-α (TNF-α) were identified in the synovial fluid of patients with OA. In chondrocytes, the overexpression of aggrecanase-2 (ADAMTS5) and matrix metalloproteinases (MMPs) by IL-1α reduced the synthesis of extracellular matrix components such as Collagen and Aggrecan (ACAN) [5]. Moreover, IL-1α has been shown to induce the overproduction of inducible nitric oxide synthase (iNOS) and cyclooxygenase-2 (COX-2), which is directly relevant to the secretion of prostaglandin E_2_ (PGE_2_) and nitric oxide (NO) [6]. The elevated levels of catabolic enzymes, prostaglandins, NO and other markers in OA fluids and tissues appear to be related to elevated levels of IL-1β IL-6, and TNF-α. Genes encoding pro-inflammatory molecules like iNOS, COX-2, IL-1β, IL-6, and TNF-α were regulated by nuclear factor kappa-light-chain-enhancer of activated B cells (NF-κB), an essential transcription factor [7]. The role of higher formations of NO and PGE_2_ have been studied as significant contributors to the development of OA. Various studies have documented that stimulation of interleukin-1α (IL-1α) and -1β (IL-1β) may potentially increase the expression of COX-2 and iNOS and consequently leads to a higher production of NO and PGE_2_ [8]. NF-κB and mitogen-activated protein kinases (MAPKs) are two major signaling pathways in the pathogenesis of OA. NF-κB activation arbitrates the response to critical pro-inflammatory cytokines, such as TNF-α, IL-1β, IL-6, and IL-8, as well as to mechanical signals and fibronectin fragments [7]. Recent studies have demonstrated that the MAPK signaling pathway was involved in the regulation of NF-κB activation and has also been reported to be involved in cytokine production [9,10]. MAPK activation is also involved in the process of cartilage degradation induced by elevated aggrecanases and MMPs [11]. Targeting the inhibition of NF-κB and MAPKs will be a deliberate objective in the treatment of OA.

Human adipose tissue-derived stem cells (ADSCs) have been introduced to replace multipotent stem cells, and are therefore considered ideal regenerative therapies, and indicated for the treatment of OA, and cardiovascular disorders [12]. ADSCs are gaining a reputation as a disease-modifying treatment because of their affluence of harvesting, safety, and potential to differentiate into cartilage tissue. ADSCs are also known for their potent anti-inflammatory and angiogenic properties, and ADSCs were investigated in wound-healing process [13,14]. Interestingly, stem cells potentially secrete a comprehensive range of trophic mediators that exert paracrine effects on other cell types. Secretion of regulatory factors determines the regenerative and therapeutic outcomes in a wide range of applications. Therefore, stem-cell-conditioned media suggest possible therapeutic applications in myocardial and osteogenic regeneration or in wound healing. Basic research studies investigating cell-mediated therapies suggest a significant role of stem cells as therapeutic tools.

In the current study, we scrutinized the potential of membrane-free stem cell components (MFSCC) derived from basal ADSCs to inhibit the inflammation stimulated by IL-1α in rat primary chondrocytes. In addition, we determined whether MFSCC have prospective chondrocyte protective effects on monosodium iodoacetate (MIA)-induced OA in rats. Appropriate mediators of articular degeneration using have been investigated using in vitro and in vivo rat models of OA to study inflammatory and cartilage degradative responses.

## 2. Results

### 2.1. Cytotoxic Effect of Membrane-Free Stem Cells Components (MFSCC) on Chondrocytes

**Figure 1:**
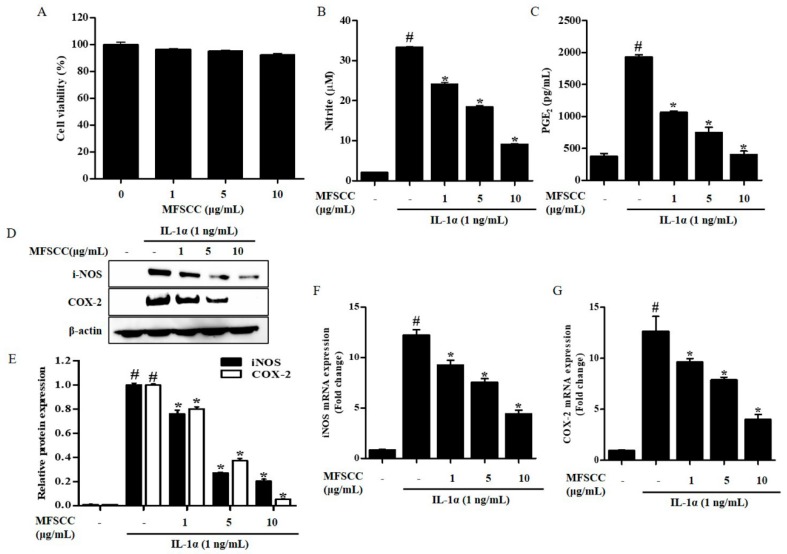
Effect of MFSCC on cell viability and inflammation in rat primary chondrocytes. (**A**) The cytotoxic effects of MFSCC were examined by CCK-8 assay and cell cultures were treated with different concentrations of MFSCC (0, 1, 5, and 10 μg/mL) for 24 h. The MFSCC showed no cytotoxicity in rat primary chondrocytes. The results are indicated as mean ± SEM (* *p* < 0.05 compared with the control group). (**B**,**C**) Anti-inflammatory equities of MFSCC (0, 1, 5, and 10 μg/mL) were determined by the estimation of NO and PGE_2_ production. (**D**–**G**) The expression of iNOS and COX-2 was detected by western blot and qRT-PCR. Rat primary chondrocytes were co-treated with different concentrations of MFSCC (0, 1, 5, and 10 μg/mL) and 1 ng/mL IL-1α for 24 h. The results are expressed as mean ± SEM (# *p* < 0.05 compared with the control group; * *p* < 0.05 compared with the IL-1α group).

To determine the cytotoxicity of MFSCC on rat primary chondrocytes, cell viability was evaluated at various concentrations of MFSCC (0, 1, 5, and 10 μg/mL) using the CCK-8 assay after incubating the cells for 24 h. The results showed that MFSCC concentration ranging from 0–10 μg/mL were not cytotoxic to rat primary chondrocytes (Figure 1A). All these three doses were considered safe and used for further experiments.

### 2.2. Effect of MFSCC on IL-1α-Induced Production of NO and PGE_2_

To examine the inhibitory effect of MFSCC on NO production in IL-1α-stimulated rat primary chondrocytes, the Griess assay was used to measure NO production. The specific release of NO after incubation with indicated concentrations of MFSCC was measured in IL-1α-stimulated primary chondrocytes and results show that MFSCC inhibited NO release in a dose-dependent manner (Figure 1B). PGE_2_ plays an important role in OA progression, and therefore its production was measured in rat primary chondrocytes. Exposure to IL-1α remarkably elevated the expression of PGE_2_. The inhibitory effect of MFSCC on PGE_2_ synthesis in IL-1α-stimulated rat primary chondrocytes was similar to its effect on NO inhibition, where MFSCC inhibited IL-1α-stimulated PGE_2_ production in a dose-dependent manner (Figure 1C).

### 2.3. Effects of MFSCC on iNOS and COX-2 Expression in Rat Primary Chondrocytes

PGE_2_ is obtained in a reaction catalyzed by COX-2. NO is an important factor mediating OA progression during inflammation and is generated by iNOS in chondrocytes. Thus, we measured the protein expression of COX-2 and iNOS in IL-1α-stimulated primary chondrocytes with or without treatment of MFSCC by using western blot and qRT-PCR, respectively. Rat primary chondrocytes stimulated with IL-1α had high iNOS and COX-2 protein and mRNA levels when compared with those of the control. The expression of iNOS and COX-2 protein expression decreased significantly in the chondrocytes after MFSCC treatment at both mRNA and protein levels. These results indicate that IL-1α-stimulated iNOS and COX-2 protein levels were suppressed in the chondrocytes treated with MFSCC (Figure 1D,G). The altered expression of COX-2 and iNOS was consistent with PGE_2_ and NO production, which was triggered by IL-1α and restored dose-dependently by MFSCC.

### 2.4. MFSCC Inhibited MMPs and Produced Cartilage Matrix Components in Il-1α-Stimulated Rat Chondrocytes

**Figure 2:**
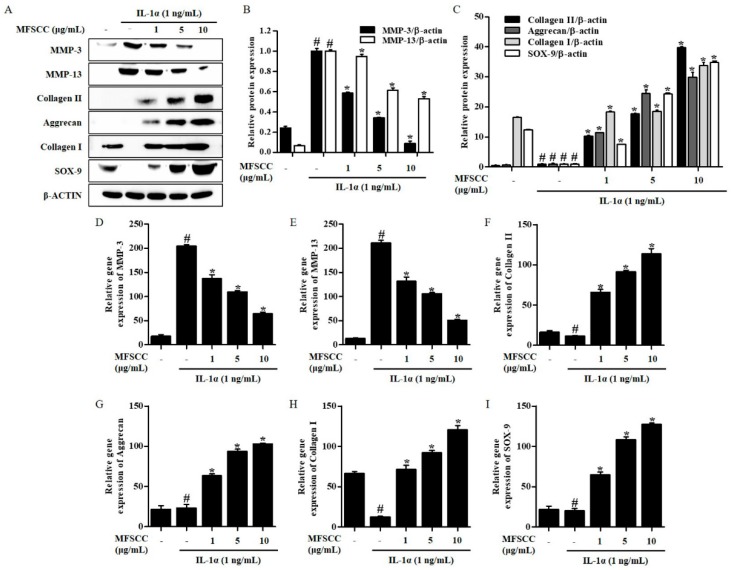
MFSCC suppressed catabolic enzymes production in IL-1α-stimulated primary chondrocytes. The rat primary chondrocytes were treated with varying concentrations of MFSCC (0, 1, 5, and 10 μg/mL) for 24 h followed by IL-1α (10 ng/mL) stimulation for 1 h. The expression of MMP-3, MMP-13, collagen II, aggrecan, collagen I, and SOX-9 was detected by western blot (**A**–**C**) and qRT-PCR (**D**–**I**). The data are expressed as mean ± SEM (# *p* < 0.05 compared with the control group; * *p* < 0.05 compared with the IL-1α group).

We investigated the effects of MFSCC on IL-1α-stimulated MMPs expression. The results revealed that IL-1α significantly elevated the synthesis of MMP-3 and MMP-13 compared with the untreated group of cells. However, MFSCC had a dose-dependent inhibitory effect on MMP-3 and MMP-13 production in IL-1α-stimulated rat primary chondrocytes (Figure 2A–E). Cartilage matrix is mainly composed of structural macromolecules such as collagen types I and II, and aggrecan. We evaluated the levels of protein and gene expression of MMPs, collagens, aggrecan, and SOX-9 in IL-1α-stimulated rat primary chondrocytes, to determine whether MFSCC attenuated cartilage degeneration. The results showed that IL-1α undoubtedly promotes the degradation of collagen types I and II, aggrecan, and SOX-9 compared with the control group. Nevertheless, MFSCC significantly induced synthesis of collagen types I and II, and aggrecan in IL-1α-stimulated rat primary chondrocytes (Figure 2A,C,F–I).

### 2.5. MFSCC Regulates the Nuclear Translocation of Nf-κB p65 the Expression of MAPKs in the Primary Chondrocytes Stimulated by IL-1α

**Figure 3:**
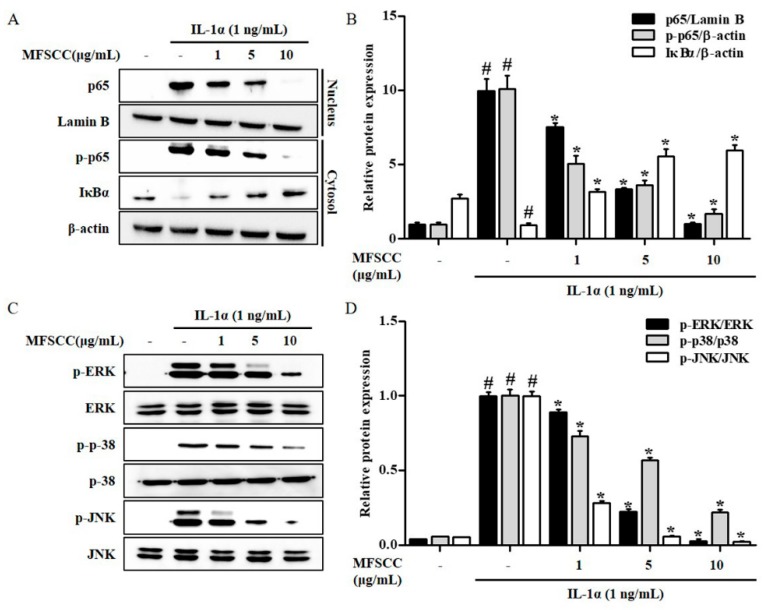
MFSCC inhibited IL-1α-stimulated phosphorylation of NF-κB and MAPKs signaling pathway. (**A**,**B**) The protein levels of p65, p-p65, and IκBα were detected by western blot. (**C**,**D**) The levels of phosphorylated ERK, p38, and JNK were determined by western blot. The rat primary chondrocytes were co-treated with MFSCC (0, 1, 5, and 10 μg/mL) and 1 ng/mL IL-1α for 24 h. The data are indicated as mean ± SEM (# *p* < 0.05 compared with the control group; * *p* < 0.05 compared with the IL-1α group).

We analyzed the effects of MFSCC on IL-1α-stimulated degradation and phosphorylation of IκB by western blot analysis in rat primary chondrocytes. The IκB levels were decreased significantly subsequent to IL-1α-stimulation. The role of transcription factor NF-κB was analyzed in IL-1α-stimulated response. The phosphorylation of IκB was inhibited by the stimulation of IL-1α, which leads to the activation of NF-κB via nuclear translocation of p65. However, MFSCC suppressed the nuclear translocation of p65 but increased its cytoplasmic fractions. These results indicate that MFSCC increased IκB protein levels via IκB dephosphorylation and p65 translocation to the cytoplasm from the nucleus in IL-1α-stimulated primary chondrocytes (Figure 3A,B). MAPKs pathways play an important role in regulating anti-inflammation in several chronic inflammatory diseases. Hence, it is important to determine whether MFSCC treatment regulated the phosphorylation of ERK, p38, and JNK in IL-1α-stimulated primary chondrocytes. MFSCC treatment for 24 h downregulated ERK, p38, and JNK in IL-1α-stimulated primary chondrocytes indicating its anti-inflammatory effect via MAPKs regulation in rat primary chondrocytes (Figure 3C,D).

### 2.6. Inhibition of Cytokines Expression by MFSCC

**Figure 4:**
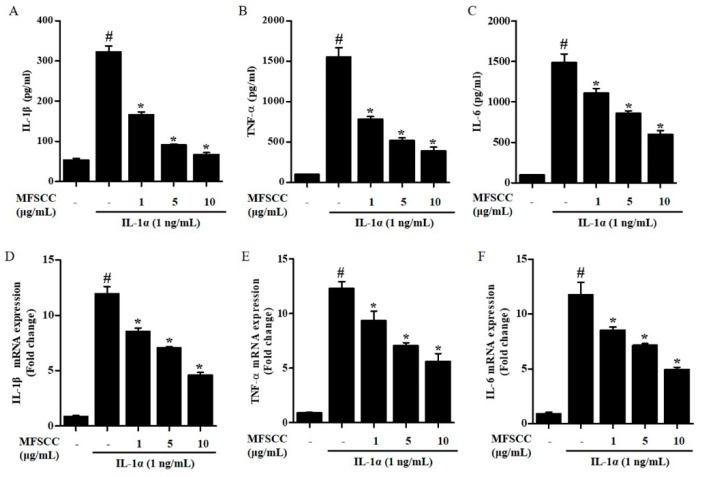
MFSCC attenuated the expression of pro-inflammatory cytokines in IL-1α-stimulated primary chondrocytes. The expression of IL-1β, TNF-α, and IL-6 was investigated by ELISA (**A**–**C**) and qRT-PCR (**D**–**F**). Rat primary chondrocytes were co-treated with MFSCC (0, 1, 5, and 10 μg/mL) and 1 ng/mL interleukin-1α for 24 h. The data results indicated as mean ± SEM (# *p* < 0.05 compared with the control group; * *p* < 0.05 compared with the IL-1α group).

Excessive synthesis of inflammatory cytokines such as IL-1β, IL-6, and TNF-α contributes to chronic inflammatory diseases. Therefore, we investigated whether MFSCC inhibited pro-inflammatory cytokines in IL-1α-stimulated chondrocytes by ELISA and qRT-PCR. The cytokine levels increased significantly following IL-1α treatment compared with those in the untreated control group. ELISA and qRT-PCR analysis results revealed that the IL-1β, IL-6, and TNF-α expression decreased in IL-1α-stimulated primary chondrocytes co-treated with the MFSCC in both protein and mRNA levels. These results show that MFSCC suppressed cytokines at the mRNA transcription and protein expression in IL-1α-stimulated chondrocytes in a dose-dependent manner (Figure 4A–F).

### 2.7. MFSCC Reduced Biomarkers of Chondrocyte in OA Rat Model

**Figure 5:**
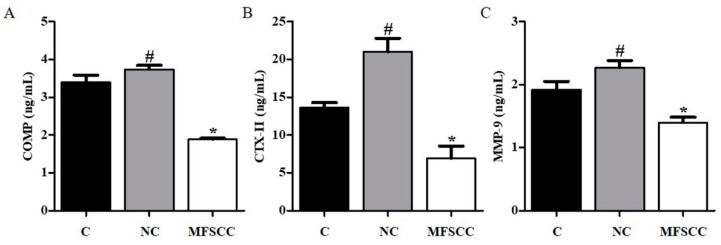
Biomarkers of cartilage degradation in a rat OA model. Expression of MMP-9, CTX-II, and COMP-2 in rat serum was analyzed by ELISA in a rat OA model (**A**–**C**). (C: injected with saline, NC: injected with MIA, and MFSCC: after 2 weeks of MIA injection, injected with MFSCC once a week for 3 weeks) The data are indicated as mean ± SEM (# *p* < 0.05 compared with the control group; * *p* < 0.05 compared with the NC group).

Next, we used an OA rat model to measure the efficacy of MFSCC in reducing the biomarkers of OA. To investigate whether MFSCC induced regeneration of articular cartilage in the selected animal rat model, we injected them into OA rats once a week after 2 weeks of MIA injection, and collected the serum samples from each group after 3 weeks of treatment. We detected serum biomarkers from rats in each group using ELISA. The MMP-9, COMP, and CTX-II expression levels increased over 3 weeks after negative product MIA injection. The expressions of MMP-9, COMP, and CTX-II were significantly reduced in MFSCC treatment (abbreviation, MFSCC) group when compared with the control (abbreviation, C) and negative control (abbreviation, NC) groups. The levels of MMP-9, COMP, and CTX-II were reduced from 2.27 to 1.4 ng/mL, from 3.73 to 1.89 ng/mL, and from 20.99 to 1.4 ng/mL in each of the NC and MFSCC groups by MFSCC treatment. These results show that MFSCC reduced the expression of MMP-9, COMP, and CTX-II of biomarker of chondrocyte increased by MIA in vivo (Figure 5). 

## 3. Discussion

OA is the most common and chronic degenerative musculoskeletal diseases associated with aging and obesity. OA is characterized by the subchondral bone sclerosis, degeneration of articular cartilage, and synovitis [3]. The pathophysiology of OA involves several convoluted pathological pathways, and inflammation is a key mechanism. Synovial cells and chondrocytes in the cartilage are affected by inflammation triggers, which release cytokines including IL-1β, TNF-α, and IL-6 and elevate the production of catabolic agents such as proteinases [5]. IL-1α is a pro-inflammatory cytokine, which plays a crucial role in bovine and human articular cartilage [15]. In addition, IL-1α is liberated in an active form upon cell damage and able to activate IL-1β and product other cytokines in progressive of OA [16]. Currently, studies focusing on stem cell-based therapy or developing alternative sources of stem cells for OA treatment, regenerate cartilage and attenuate on-going inflammation [17,18]. ADSC therapy is a stem cell-based approach with potential therapeutic application for chronic diseases and is mediated via anti-inflammatory, anti-apoptotic, pro-trophic, and pro-angiogenic effects [19]. In our recently published article, we have shown the anti-inflammatory effect of MFSCC on RAW 264.7 macrophages cells by reducing NO production, and also inhibiting iNOS and COX-2 (the publication is not online, but was accepted in “Evidence-Based Complementary and Alternative Medicine”). What remains to be explored is whether the components of these stem cells possess an anti-inflammatory effect, and the detailed mechanism to confirm its role in OA therapy. We investigated whether the novel components of MFSCC from ADSCs have an effect on anti-inflammation and cartilage regeneration in IL-1α-stimulated rat primary chondrocytes and a rat model of OA.

In the present study, we have demonstrated that MFSCC inhibit IL-1α-stimulated inflammation in vitro and reduced the degradation of cartilage factors in vivo. Our data showed that IL-1α significantly up-regulated the inflammatory factors, including the expression of iNOS, COX-2, NO, and PGE_2_. Moreover, IL-1α considerably induced the production of cytokines such as TNF-α, IL-1β, and IL-6. Co-treatment with MFSCC significantly inhibited the levels of inflammatory factors and cytokines in IL-1α-stimulated primary chondrocytes, primarily indicating its anti-inflammatory effect in OA.

In the pathogenesis of OA, IL-1α induces the expression of catabolic enzymes such as MMPs, COMP, and CTX-II that plays a vital role in the degradation of cartilage and also serves as a biomarker of OA [20,21]. First, we investigated the MFSCC-regulated MMPs in IL-1α-stimulated rat primary chondrocytes in vitro. MMPs participate in the decomposition of extracellular matrix such as collagens and aggrecan in normal physiological processes [22]. In our study, the levels of MMP-3 and MMP-13 were increased by IL-1α in primary chondrocytes, whereas co-treatment with MFSCC inhibited IL-1α-stimulated MMP-3 and MMP-13 protein expression. Furthermore, IL-1α stimulated the down-regulation of collagen types I and II and aggrecan, which was alleviated by MFSCC in vitro. In addition, we measured the concentration of catabolic enzymes in serum obtained from a rat model of OA. MIA-induced OA in rat model increased the levels of MMP-9, cartilage oligomeric matrix protein (COMP), and C-telopeptide of type II collagen (CTX-II). Proteolytic fragments of COMP have been detected in synovial fluid, degenerating cartilage, and serum of patients with knee injuries, primary and post-traumatic OA. COMP is a presumptive substrate for MMPs, including MMP-13. The degeneration products of type II collagen have been playing a critical role in OA and CTX-II is one of the most studied markers among degradation products of several type II collagens [22,23,24,25]. Studies have demonstrated elevated levels of CTX-II in OA patients compared with a non-OA population [26,27]. Nevertheless, these increased catabolic enzymes are reduced by the MFSCC treatment in rat OA model indicating its anti-inflammatory effect to improve OA condition in vivo.

Since inflammatory and cartilage degradation processes play a major role in the damage of articular tissues, many in vitro and in vivo studies have scrutinized the contribution of components of the NF-κB signaling pathways to the pathogenesis of OA [28,29]. Enhanced nuclear localization of NF-κB will lead to the over-production of NO, which accelerates inflammation, and plays a decisive role in OA by releasing numerous inflammatory factors as well as MMPs [25]. The down-stream NF-κB was not translocated to the nucleus, and remained in the cytoplasm bound to its inhibitor IκBα in the IL-1α-stimulated primary chondrocytes treated with MFSCC. Recently, several studies have shown the relationship of MAPK studies alleviating OA. Activation of MAPKs is involved in the process of inflammation induced by elevated production of cytokines [30]. In our study, IL-1α induced the phosphorylation of ERK, p-38, and JNK. However, MFSCC suppressed the activation of MAPK signaling pathway. These results indicated that the anti-inflammatory and anti-catabolic effect of MFSCC might be mediated via MAPK signaling pathway and suppression of NF-κB nuclear translocation. Our results, however, have certain limitations, because in the current study we utilized stem cells from a single source to prepare MFSCC, thus lacking a heterogeneous sample. Further studies need to be performed to confirm these data using multiple sources of MFSCC samples in the context of different age, race, and body type.

## 4. Material and Methods

### 4.1. Chemicals and Reagents

Dulbecco’s modified Eagle’s medium (DMEM), phosphate-buffered saline (PBS), fetal bovine serum (FBS), and antibiotics penicillin/streptomycin (P/S) were purchased from Gibco (BRL Life Technologies, Grand Island, NY, USA). Antibodies to iNOS, COX-2, MMP-3, NF-κB, IκBα, ERK, p-EKR, p38, p-p38, JNK, and p-JNK were purchased from Cell Signaling Technology (Danvers, MA, USA). MMP-13, collagen types I and II, and aggrecan were obtained from Novus Biologicals Inc., (Littleton, CO, USA). Lamin B and β–actin antibody were obtained from Santa Cruz Biotechnology (Santa Cruz, CA, USA) and Sigma-Aldrich (St. Louis, MO, USA).

### 4.2. Preparation of Membrane-Free Stem Cell Components (MFSCC)

The membrane-free stem cell components (MFSCC) used in this test were produced using patented technology. MFSCC was composed of stem cell components, after separating the stem cell membranes and culturing them from body fat tissue. The fat tissue was provided by a healthy female in her twenties with a BMI of 25 to 29.9 (second degree obesity) considered appropriate based on blood tests and physician diagnosis. The blood was tested for viruses including hepatitis B virus (HVB), hepatitis C virus (HCV), human immunodeficiency virus (HIV), human T-cell lymphocytic virus (HTVL), parvovirus B19, cytomegalovirus (CMV), epstein-barr virus (EBV), and *Treponema pallidum*. The donor provided written informed consent and the Regional Committee on Biomedical Research Ethics approved the clinical protocol. Visceral adipose tissue, which were screened for safety as mentioned above, were separated and purified. The cells were cultured at 37 °C and 5% CO_2_, in a standard incubator using serum-free cell culture medium. After the cell growth reached 70–80% confluence, the cells were sub-cultured until 6 to 8 passages. ADSC cells were characterized using specific markers (positive markers: CD105, CD29: negative maker: CD34) by an immunofluorescence assay (data not shown). A certain amount of stem cells was collected; the cell membranes were removed by ultra sonication, and eliminate the debris of the membranes by centrifugation at 800–1500 g, following successive filtration. The aqueous solution of MFSCC was further lyophilized and stored in powder form. Membrane-free stem cells (MFSCs) were obtained as the final product, and found non-toxic based on nine safety tests conducted by accreditation authority certified under Good Laboratory Practice (GLPs). A schematic diagram of MFSCC preparation has shown in Figure S1.

### 4.3. Primary Chondrocyte Culture

Primary chondrocytes were isolated from the cartilage of 11-week-old Wistar rats. Immediately, the cartilage was rinsed three times in PBS. The cartilage was sectioned into fragments measuring about 0.5–1 mm^3^. The sectioned tissues were placed in 100 mm dishes containing DMEM medium without FBS and P/S at 37 °C in a 5% CO_2_ incubator. When chondrocytes leak out of the sectioned tissues and adhered to the dish, the tissue was removed, and the chondrocytes were washed with PBS. A vital cell count was performed by trypan blue exclusion, and the 2.5 × 10^6^ cells were seeded in each 100 mm dish in DMEM medium supplemented with 10% FBS and 1% P/S at 37 °C in a 5% CO_2_ incubator.

### 4.4. Cell Viability Assay

To determine potential cytotoxic effects of MFSCC on primary chondrocyte stimulated with IL-1α, the cell viability was measured by Cell Counting Kit-8 (CCK-8, Dojindo Molecular Technologies, Inc.) in the absence or presence of MFSCC. The primary chondrocytes were seeded at a density of 2.5 × 10^4^ cells/well in 96-well plates and incubated for optimal growth at 37 °C for 24 h. The optimally grown cells pre-treated with or without IL-1α (1 ng/mL) followed by treatment with various concentrations of MFSCC (0, 1, 5, and 10 μg/mL) were incubated at 37 °C for 24 h. After 24 h, 10 μL of CCK-8 solution was added to each well, followed by further incubation for 2 h at 37 °C. The color was subsequently analyzed at 450 nm using a microplate reader (BioTek Instruments, Inc., Winooski, VT, USA). Cell proliferation was calculated as a percentage compared with the positive and negative control groups accordingly, which was set at 100%.

### 4.5. NO Production Assay

The primary chondrocytes were plated in 96-well plates (2.5 × 10^4^ cells/well) and treated with MFSCC stimulated with IL-1α for 24 h. The quantity of nitrite formed from NO was measured according to the manufacturer’s protocol. Briefly, the nitrites were quantified by mixing 150 μL culture medium diluted in 130 μL D.W. with a 20 μL Griess reagent (1% sulfanylamide, 0.1% naphthylethylene diamine, and 2.5% H_3_PO_4_). Reaction mixtures were incubated for 30 min at room temperature and the reaction was stopped by adding the stop solution, and the optical density was read at 548 nm.

### 4.6. Enzyme-Linked Immunosorbent Assay (ELISA)

Cell culture supernatants were collected and assayed for cytokines. The levels of prostaglandin E2 (PGE_2_), interleukin-1β (IL-1β), tumor necrosis factor-α (TNF-α), interleukin-6 (IL-6), and MMP-9 were quantified using enzyme-linked immunosorbent assay (ELISA) kits (R&D Systems, Minneapolis, MN, USA). The levels of cross-linked C-telopeptide of type II collagen (CTX-II) and cartilage oligomeric matrix protein (COMP) were quantified using MyBioSource Inc. ELISA kits (San Diego, CA, USA). Experiments were performed as instructed by the respective kit manuals.

### 4.7. Western Blot Analysis

For western blot analysis, the primary chondrocytes were treated with indicated concentrations of MFSCC for 24 h and cells were lysed in ice-cold M-PER buffer. The nuclear and cytoplasmic fractions were separated using Nuclear and Cytoplasmic Extraction Reagents (NE-PER, Thermo Scientific, MO, USA). The protein concentrations were determined using Bradford assay (Bio-rad, CA, USA). The membranes were blocked with 5% non-fat skimmed milk in Tris-buffered saline containing 1% Tween 20 (TBS-T, pH 7.4) at room temperature for 1 h, and incubated overnight at 4 °C with a 1:1,000 dilution of respected primary antibody. The membranes have washed five times with TBS-T for 10 min each at room temperature, incubated with a 1:2000 dilution of HRP conjugated secondary antibody for 3 h at room temperature. Blots were developed under an ECL detection system (Bio-Rad, Hercules, CA, USA). The bands were quantitatively analyzed by using the Image Lab^TM^ version 6.0.1 software (Bio-Rad, Hercules, CA, USA). The densitometry readings of the bands were normalized according to β-actin expression.

### 4.8. qRT-PCR

Trizol reagent (GeneALL, Biotechnology, Seoul, Korea) was used for isolating total RNA, according to the manufacture’s protocol after treated with indicated concentrations of MFSCC for 24 h at 37 °C. Obtained total RNA was quantified using a NanoDrop spectrophotometer and were reverse-transcribed at 42 °C into cDNA for 30 min using QuantiTect Probe PCR kit (Qiagen, GmbH, Hilden, Germany). The cDNA was successively amplified by PCR using the PCR Master Mix (Promega, Madison, WI, USA) according to the manufacturer’s instructions in a Thermal Cycler Dice^®^ Real-Time PCR system (TaKaRa Bio, Kyoto, Japan). Relative fold levels were measured using β-actin genes as normalizer control. The primer was purchased from Bioneer (Seoul, Korea) (Table 1).

### 4.9. Animals

Wistar rats were procured from Orientbio (Seongnam, Korea), and fed with pellets and water ad libitum. The animal experimental protocol was reviewed and approved by the Korea Institute of Toxicology Gyeongnam Department of Environmental Toxicology and Chemistry Institutional Animal Care and Use Committee (No.1808-0004, 06/12/2018).

All animals were housed in a room at temperature ranging from 20 °C to 27 °C, a relative humidity 40–60%, under approximately 12 h light/12 h dark cycle and ventilation frequency of 10 to 20 times each hour. Animals were acclimated to the laboratory environment for 1 week prior to experimental treatment. This study was reviewed and assessed by the Korea Institute of Toxicology Gyeongnam Department of Environmental Toxicology and Chemistry Institutional Animal Care and Use Committee (1808-0004).

### 4.10. Induction of OA in Rats

After 1 week of acclimation, rats were anesthetized with isoflurane and administered an intra-articular injection of 3 mg/kg MIA (Sigma-Aldrich) to the right knee using a 30G needle in 50 μL volume. The normal control group was injected with 0.9% saline (JW Pharmaceutical Co., Seoul, Korea). After 2 weeks of MIA injection, animals were divided into three groups of five animals each: normal, negative, and treatment (40 μg/head). All animals were intramuscularly treated to the right femur proximal once a week for 3 weeks. Blood collected from each group was analyzed for further analysis.

### 4.11. Statistical Analysis

Differences between the groups were examined for statistical significance with Student’s t-test. One-way ANOVA test using GraphPad Prism version 5.01 (San Diego, CA, USA) was used for data analysis. The results are expressed as the mean ± standard deviation (SD) of at least three independent experiments. A value of *p* < 0.05 was considered significant.

## 5. Conclusions

Overall, the results suggest that MFSCC decreased the expression of inflammatory cytokines such as TNF-α, IL-1β, and IL-6 in IL-1α-stimulated primary rat chondrocytes. Furthermore, MFSCC reduced the expression of MMP-9, COMP, and CTX-II in the serum of MIA-induced OA rat model. The results suggest that MFSCC suppress NF-κB and MAPKs-mediated inflammatory pathways in vitro. In addition, the results demonstrate that MFSCC prevents cartilage degradation in vivo. To the best of our knowledge, this is the first study to elucidate the anti-inflammatory and anti-catabolic effects of MFSCC both in vitro and in vivo. Our current results provide primary evidence for considering MFSCC as a novel non-cell-based stem cell therapeutic component for treatment of chronic inflammatory diseases such as OA.

## Figures and Tables

**Table 1:** Sequences of the primers used for qRT-PCR.

Primers	Forward Primer (5′→3′)	Reverse Primer (5′→3′)
iNOS	TGGATGCAACCCCATTGTC	CCCGCTGCCCCAGTTT
COX-2	CAAATCCTTGCTGTTCCCACCCAT	GTGCACTGTGTTTGGAGTGGGTTT
MMP-3	CTCTGGAGTAATGTCACACCTCT	TGTTGGTCCACCTTTCATCTTC
MMP-13	ACGTTCAAGGAATCCAGTCTCTCT	GGATAGGGCTGGGTCACACTT
SOX-9	CATCAAGACGGAGCAACTGAG	GTGGTCGGTGTAGTCATACTGC
Collagen II	TCAACAATGGGAAGGCGTGAG	GTTCACGTACACTGCCCTGAAG
Aggrecan	GGGCGTCAGAACTGTCTACC	ACTGACACACCTCGGAAGC
Collagen I	AGCGCTGGTTTCGACTTCAGCTTCC	CATCGGCAGGGTCGGAGCCCT
IL-1β	TGATGGCTTATTACAGTGGCAATG	GTAGTGGTGGTCGGAGATTCG
TNF-α	ATCTTCTCGAACCCCGAGTG	GGGTTTGCTACAACATGGGC
IL-6	GTGTTGCCTGCTGCCTTC	AGTGCCTCTTTGCTGCTTTC

## Supplementary Materials

Supplementary materials can be found at https://www.mdpi.com/1422-0067/20/19/4869/s1.
