# Supplementary material for: Membrane-Free Stem Cell Components Inhibit Interleukin-1α-Stimulated Inflammation and Cartilage Degradation In Vitro and In Vivo: A Rat Model of Osteoarthritis
Source: Int J Mol Sci. 2019 Sep 30;20(19):4869. doi: 10.3390/ijms20194869 (PMC6801847; doi:10.3390/ijms20194869)
Supplement: Supplementary file 1 [file ijms-20-04869-s001.pdf]

Membrane-Free Stem Cell Components Inhibit Interleukin-1α-Stimulated Inflammation and Cartilage Degradation in vitro and in vivo: A Rat Model of Osteoarthritis

Ho Jeong Lee, Seon Min Lee, Yeon Gyu Moon, Yeon Seop Jung, Ju Hong Lee, Venu Venkatarame Gowda Saralamma, Young Sil Kim, Jung Eun Pak, Hye Jin Lee, Gon Sup Kim and Jeong Doo Heo

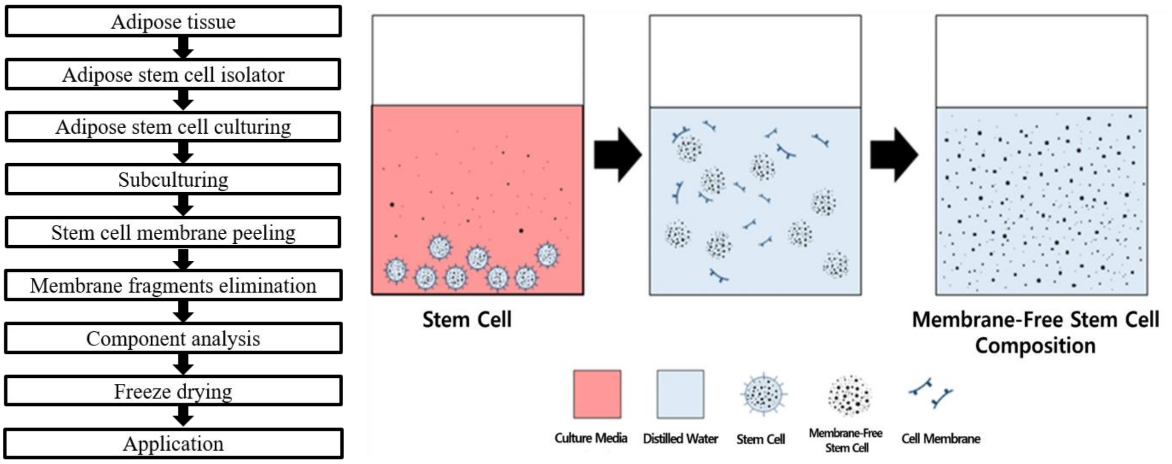

Figure S1. A schematic diagram of MFSCC preparation.
